# 2,2′-Bi-1,3,4-thia­diazole-5,5′-diamine tetra­hydrate

**DOI:** 10.1107/S160053681002996X

**Published:** 2010-08-04

**Authors:** Chaveng Pakawatchai, Saowanit Saithong

**Affiliations:** a Department of Chemistry and Centre for Innovation in Chemistry, Faculty of Science, Prince of Songkla University, Hat Yai, Songkhla 90112, Thailand

## Abstract

In the title compound, C_4_H_4_N_6_S_2_·4H_2_O, the complete organic mol­ecule is generated by crystallographic twofold symmetry and the dihedral angle between the aromatic rings is 10.24 (3)°. In the crystal, inter­molecular N—H⋯N, N—H⋯O, O—H⋯N and O—H⋯O hydrogen bonds and aromatic π–π stacking inter­actions [centroid–centroid separations = 3.530 (3) and 3.600 (3) Å] are observed.

## Related literature

For background to the pharmacutical properties of thia­dia­zo­les, see: Chapleo *et al.* (1986 ▶; 1987 ▶); Stillings *et al.* (1986 ▶); Clerici *et al.* (2001 ▶). For their tribological behavior, see: Zhu *et al.* (2009 ▶) and for their pesticidal activity, see: Fan *et al.* (2010 ▶).
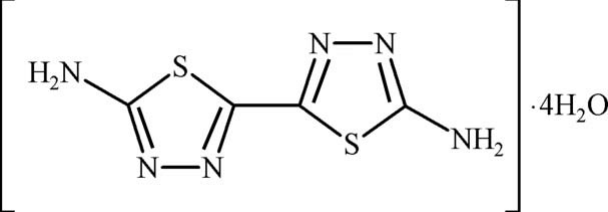


## Experimental

### 

#### Crystal data

C_4_H_4_N_6_S_2_·4H_2_O
*M* *_r_* = 272.32
Monoclinic, 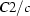

*a* = 19.977 (6) Å
*b* = 6.678 (2) Å
*c* = 9.328 (3) Å
β = 112.514 (6)°
*V* = 1149.7 (6) Å^3^
*Z* = 4
Mo *K*α radiation
μ = 0.48 mm^−1^
*T* = 293 K
0.29 × 0.06 × 0.03 mm

#### Data collection

Bruker APEX CCD diffractometer
Absorption correction: multi-scan (*SADABS*; Bruker, 2003 ▶) *T*_min_ = 0.659, *T*_max_ = 1.000
5047 measured reflections
1015 independent reflections
902 reflections with *I* > 2σ(*I*)
*R*_int_ = 0.040

#### Refinement

*R*[*F* ^2^ > 2σ(*F* ^2^)] = 0.034
*wR*(*F* ^2^) = 0.091
*S* = 1.12
1015 reflections
91 parameters
6 restraints
H atoms treated by a mixture of independent and constrained refinement
Δρ_max_ = 0.36 e Å^−3^
Δρ_min_ = −0.29 e Å^−3^

### 

Data collection: *SMART* (Bruker, 1998 ▶); cell refinement: *SAINT* (Bruker, 2003 ▶); data reduction: *SAINT*; program(s) used to solve structure: *SHELXS97* (Sheldrick, 2008 ▶); program(s) used to refine structure: *SHELXL97* (Sheldrick, 2008 ▶); molecular graphics: *Mercury* (Macrae *et al.*, 2008 ▶); software used to prepare material for publication: *Mercury*.

## Supplementary Material

Crystal structure: contains datablocks I, global. DOI: 10.1107/S160053681002996X/hb5580sup1.cif

Structure factors: contains datablocks I. DOI: 10.1107/S160053681002996X/hb5580Isup2.hkl

Additional supplementary materials:  crystallographic information; 3D view; checkCIF report

## Figures and Tables

**Table 1:** Hydrogen-bond geometry (Å, °)

*D*—H⋯*A*	*D*—H	H⋯*A*	*D*⋯*A*	*D*—H⋯*A*
N3—H3*A*⋯O2^i^	0.88 (2)	2.11 (2)	2.953 (3)	161 (2)
N3—H3*B*⋯N2^ii^	0.88 (2)	2.12 (2)	2.981 (3)	170 (3)
O1—H1*A*⋯N1^iii^	0.83 (2)	2.05 (2)	2.872 (3)	171 (3)
O1—H1*B*⋯O2^iv^	0.83 (2)	1.96 (2)	2.780 (3)	168 (3)
O2—H2*A*⋯O1^v^	0.82 (2)	2.07 (2)	2.867 (3)	167 (3)
O2—H2*B*⋯O1^vi^	0.83 (2)	1.97 (2)	2.806 (3)	178 (3)

Symmetry codes: (i) 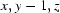; (ii) 
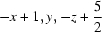
; (iii) 
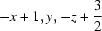
; (iv) 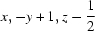; (v) 
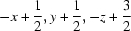
; (vi) 
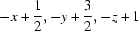
.

**Fig. 1.**
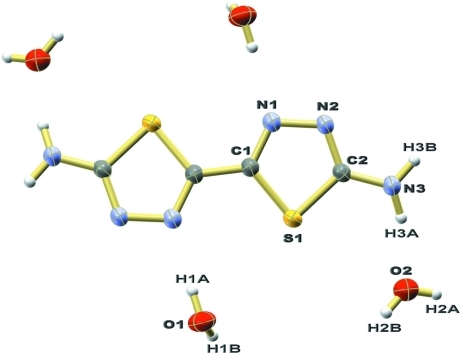
The molecular structure of (I) with displacement ellipsoids plotted at the 30% probability level. H atoms are omitted.

**Fig. 2.**
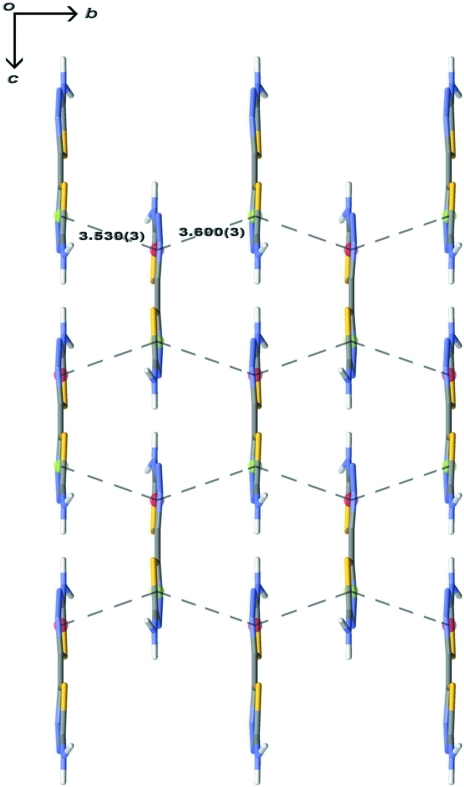
Centriod-centriod interaction distances between thiadiazoline rings in packing plot down a axis. Water layers are omitted.

**Fig. 3.**
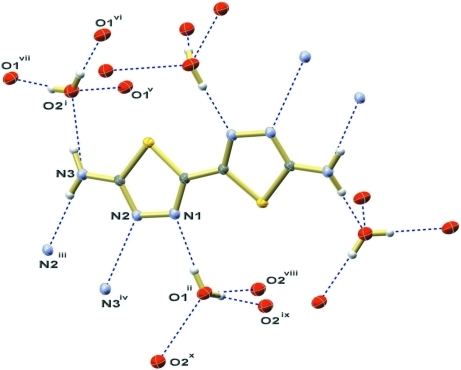
The interactions of the [5,5'-amine-(2,2'-1,3,4-thiadiazole)] tetrahydrate compound. Symmetry code: i= x, y - 1,z; ii = 1 - x,y,1.5 - z; iii = 1 - x, y 2.5 - z; iv = 1 - x, y, 2.5 - z; v = x, -y, 1/2 + z; vi = 1/2 - x, 1/2 - y, 1 - z; vii = 1/2 - x, y - 1/2, 1 - z; viii = 1 - x, 1 - y, 2 - z; ix = 1/2 + x, y - 1/2, z; x = 1/2 + x, 1.5 - y, 1/2 - z.

**Fig. 4.**
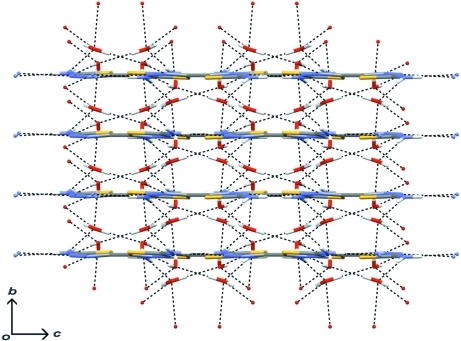
The interactions packing plot down a axis.

**Fig. 5.**
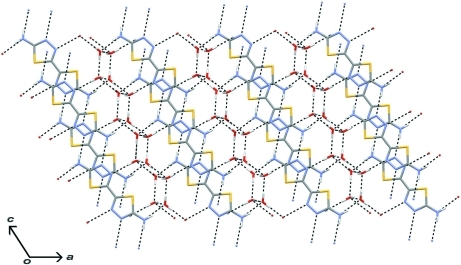
The interactions packing plot down b axis.

**Fig. 6.**
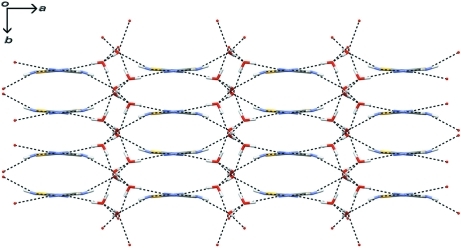
The interactions packing plot down c axis.

## supplementary crystallographic information

### Comment

Most of Shift bases, five membered heterocyclic thiadiazole derivatives containg N and S atoms were studied and reported due to the various applications. Different classes of thiadiazole compounds were found to be pharmacologically active using antihypertensive, anticonvulsant (Chapleo *et al.*, 1986; 1987; Stillings *et al.*, 1986), anti-depressant and anxiolytic activities (Clerici *et al.*, 2001). Recently, the friction and wear properties of 1,3,4-thiadiazole-2-thione derivatives have attracted a considerable amount of research effort. The compounds were synthesized and tested their tribological behavior as additive in rapeseed oil (ROS) to possess good thermal stabilities and anti-corrosive abilities and excellent load-carrying capacities. Moreover, they have good anti-wear and friction-reducing properties (Zhu *et al.*, 2009). In the agriculture, these compounds are widely use as pesticides activities and they have been comercialed as agrochemicals (Fan *et al.,* 2010)

In the present work, the title compound is the by-product of the reaction between copper(I) thiocyanate and 5-amino-1,3,4-thiadiazole-2-thiol ligand. Its crystal structure is reported here. The compound crystallizes in monoclinic system, space group *C*2/*c*. The asymetric unit contains half the molecules of the title compound. The crystal structure consists of discrete molecules (Fig. 1) and 5,5'-amine-(2,2'-1,3,4-thiadiazole) molecules arrange as alternated sheets lying parallel to [010]. In addition, each set of sheets are separated by the layer of water molecules runing in same direction. The dihedral angle between mean plane of two thiadiazole rings is 10.24 (3)°. It could be indicated the effect of centroid-centroid interactions with the distances of 3.530 (3) and 3.600 (3)Å between the adjacent thidiazole rings in the same layer as depicted in Fig. 2. In the crystal lattice, amide nitrogen protons form N3—H3A···O2^ii^ [N3···O2^ii^ = 2.953 (3) Å] and N3—H3B···N2^iii^ [N3···N2^iii^ = 2.981 (3) Å] intermolecular hydrogen bonds with oxygen atoms of water molecules and one nitrogen atom of thiadiazole ring of adjacent molecules. While another thiadiazole nitrogen atom forms hydrogen bonding interaction with another water molecule in packing, O1—H1A···N1^i^ [O1···N1^i ^= 2.872 (3) Å]. In addition, The packing is also stabilized by the another type of hydrogen bond, O—H···O, among water molecules [O1···O2^iv^ = 2.780 (3) Å, O2···O1^v^ = 2.867 (3) Å, O2···O1^vi^ = 2.806 (3) Å; iv, v and vi are the symmetry codes as given in Table 1]. The hydrogen bonds are shown in Fig. 3. The interactions in packings are generated the three-dimensional interaction networks and the interaction views down three axes are depicted in Fig. 4, 5 and 6.

### Experimental

The 5-amino-1,3,4-thiadiazole-2-thiol (0.28 g, 2.1 mmol) was dissolved in acetronitile (40 ml) and then copper(I) thiocyanate salt (0.16 g, 1.3 mmol) was added. After that, the reaction of mixture was performed under ultrasonic activation (338–340 K, 40 kHz) for 2 h. The light yellow filtration was kept and allowed to slowly to room temperature. Colourless blocks and rods of the title compound were recovered after a few days. Insufficient crystalline material was obtained for elemental analysis.

### Refinement

All hydrogen atoms were located in a difference Fourier map and restrained to ride on their parent atoms, N—H = 0.87–0.89 Å with *U*_iso_(H) = 1.2*U*_eq_(N) and O—H = 0.81–0.84 Å with *U*_iso_(H) = 1.2*U*_eq_(O), respectively.

### Crystal data

**Table:**

C_4_H_4_N_6_S_2_·4H_2_O	*F*(000) = 568
*M**_r_* = 272.32	*D*_x_ = 1.573 Mg m^−^^3^
Monoclinic, *C*2/*c*	Mo *K*α radiation, λ = 0.71073 Å
Hall symbol: -C 2yc	Cell parameters from 1375 reflections
*a* = 19.977 (6) Å	θ = 2.2–27.5°
*b* = 6.678 (2) Å	µ = 0.48 mm^−^^1^
*c* = 9.328 (3) Å	*T* = 293 K
β = 112.514 (6)°	Rod, colourless
*V* = 1149.7 (6) Å^3^	0.29 × 0.06 × 0.03 mm
*Z* = 4	

### Data collection

**Table:**

Bruker APEX CCD diffractometer	1015 independent reflections
Radiation source: fine-focus sealed tube	902 reflections with *I* > 2σ(*I*)
graphite	*R*_int_ = 0.040
Frames, each covering 0.3 ° in ω scans	θ_max_ = 25.0°, θ_min_ = 2.2°
Absorption correction: multi-scan (*SADABS*; Bruker, 2003)	*h* = −23→23
*T*_min_ = 0.659, *T*_max_ = 1.000	*k* = −7→7
5047 measured reflections	*l* = −11→11

### Refinement

**Table:**

Refinement on *F*^2^	Primary atom site location: structure-invariant direct methods
Least-squares matrix: full	Secondary atom site location: difference Fourier map
*R*[*F*^2^ > 2σ(*F*^2^)] = 0.034	Hydrogen site location: inferred from neighbouring sites
*wR*(*F*^2^) = 0.091	H atoms treated by a mixture of independent and constrained refinement
*S* = 1.12	*w* = 1/[σ^2^(*F*_o_^2^) + (0.046*P*)^2^ + 0.8924*P*] where *P* = (*F*_o_^2^ + 2*F*_c_^2^)/3
1015 reflections	(Δ/σ)_max_ < 0.001
91 parameters	Δρ_max_ = 0.36 e Å^−^^3^
6 restraints	Δρ_min_ = −0.29 e Å^−^^3^

### Special details

**Table:**

Geometry. All e.s.d.'s (except the e.s.d. in the dihedral angle between two l.s. planes) are estimated using the full covariance matrix. The cell e.s.d.'s are taken into account individually in the estimation of e.s.d.'s in distances, angles and torsion angles; correlations between e.s.d.'s in cell parameters are only used when they are defined by crystal symmetry. An approximate (isotropic) treatment of cell e.s.d.'s is used for estimating e.s.d.'s involving l.s. planes.
Refinement. Refinement of *F*^2^ against all reflections. The weighted *R*-factor *wR* and goodness of fit *S* are based on *F*^2^, conventional *R*-factors *R* are based on *F*, with *F* set to zero for negative *F*^2^. The threshold expression of *F*^2^ > 2*σ*(*F*^2^) is used only for calculating *R*-factors(gt) *etc*. and is not relevant to the choice of reflections for refinement. *R*-factors based on *F*^2^ are statistically about twice as large as those based on *F*, and *R*- factors based on all data will be even larger.

### Fractional atomic coordinates and isotropic or equivalent isotropic displacement parameters (Å^2^)

**Table:**

	*x*	*y*	*z*	*U*_iso_*/*U*_eq_	
S1	0.40277 (3)	0.22880 (9)	0.80732 (6)	0.0302 (2)	
C1	0.49090 (11)	0.2498 (3)	0.8184 (2)	0.0254 (5)	
C2	0.43905 (11)	0.2386 (3)	1.0092 (2)	0.0250 (5)	
N1	0.53913 (9)	0.2631 (2)	0.9582 (2)	0.0275 (4)	
N2	0.51008 (10)	0.2557 (3)	1.0700 (2)	0.0278 (4)	
N3	0.39755 (11)	0.2341 (3)	1.0925 (2)	0.0346 (5)	
H3A	0.3518 (10)	0.196 (4)	1.049 (3)	0.042*	
H3B	0.4198 (14)	0.236 (4)	1.194 (2)	0.042*	
O1	0.30903 (9)	0.3504 (3)	0.3923 (2)	0.0468 (5)	
H1A	0.3539 (10)	0.338 (5)	0.434 (3)	0.056*	
H1B	0.2881 (16)	0.242 (3)	0.391 (4)	0.056*	
O2	0.25880 (9)	1.0305 (3)	0.9186 (2)	0.0451 (5)	
H2A	0.2393 (14)	0.997 (5)	0.977 (3)	0.054*	
H2B	0.2376 (14)	1.067 (4)	0.827 (2)	0.054*	

### Atomic displacement parameters (Å^2^)

**Table:**

	*U*^11^	*U*^22^	*U*^33^	*U*^12^	*U*^13^	*U*^23^
S1	0.0234 (3)	0.0449 (4)	0.0199 (3)	0.0004 (2)	0.0057 (2)	−0.0007 (2)
C1	0.0253 (11)	0.0284 (11)	0.0227 (11)	0.0003 (8)	0.0096 (9)	0.0001 (8)
C2	0.0270 (11)	0.0256 (11)	0.0206 (11)	0.0016 (8)	0.0071 (9)	−0.0001 (8)
N1	0.0273 (10)	0.0324 (10)	0.0230 (10)	0.0002 (7)	0.0100 (8)	0.0004 (7)
N2	0.0269 (9)	0.0364 (11)	0.0203 (9)	0.0005 (7)	0.0093 (8)	0.0002 (7)
N3	0.0275 (10)	0.0542 (13)	0.0232 (10)	−0.0021 (8)	0.0109 (9)	−0.0003 (9)
O1	0.0292 (9)	0.0543 (12)	0.0501 (11)	−0.0052 (8)	0.0076 (8)	0.0054 (9)
O2	0.0356 (10)	0.0546 (11)	0.0420 (11)	−0.0013 (8)	0.0113 (8)	0.0006 (9)

### Geometric parameters (Å, °)

**Table:**

S1—C1	1.729 (2)	N3—H3A	0.882 (17)
S1—C2	1.741 (2)	N3—H3B	0.875 (17)
C1—N1	1.294 (3)	O1—H1A	0.834 (18)
C1—C1^i^	1.455 (4)	O1—H1B	0.833 (17)
C2—N2	1.316 (3)	O2—H2A	0.815 (17)
C2—N3	1.337 (3)	O2—H2B	0.834 (17)
N1—N2	1.375 (3)		
			
C1—S1—C2	86.53 (10)	C2—N2—N1	111.99 (17)
C1—S1—C2	86.53 (10)	C2—N2—N1	111.99 (17)
N1—C1—C1^i^	122.9 (2)	C2—N3—H3A	120.2 (17)
N1—C1—S1	114.49 (16)	C2—N3—H3A	120.2 (17)
C1^i^—C1—S1	122.6 (2)	C2—N3—H3B	117.0 (19)
N2—C2—N3	123.9 (2)	C2—N3—H3B	117.0 (19)
N2—C2—S1	113.78 (16)	H3A—N3—H3B	121 (3)
N3—C2—S1	122.30 (17)	H1A—O1—H1B	111 (3)
C1—N1—N2	113.20 (17)	H2A—O2—H2B	126 (3)
			
C2—S1—C1—N1	−0.35 (15)	C1^i^—C1—N1—N2	−178.63 (10)
C2—S1—C1—N1	−0.35 (15)	S1—C1—N1—N2	0.6 (2)
C2—S1—C1—C1^i^	178.91 (8)	N3—C2—N2—N1	−178.00 (19)
C2—S1—C1—C1^i^	178.91 (8)	S1—C2—N2—N1	0.4 (2)
C1—S1—C2—N2	−0.02 (16)	C1—N1—N2—C2	−0.6 (2)
C1—S1—C2—N3	178.36 (19)	C1—N1—N2—C2	−0.6 (2)

Symmetry codes: (i) −*x*+1, *y*, −*z*+3/2.

### Hydrogen-bond geometry (Å, °)

**Table:**

*D*—H···*A*	*D*—H	H···*A*	*D*···*A*	*D*—H···*A*
N3—H3A···O2^ii^	0.88 (2)	2.11 (2)	2.953 (3)	161 (2)
N3—H3B···N2^iii^	0.88 (2)	2.12 (2)	2.981 (3)	170 (3)
O1—H1A···N1^i^	0.83 (2)	2.05 (2)	2.872 (3)	171 (3)
O1—H1B···O2^iv^	0.83 (2)	1.96 (2)	2.780 (3)	168 (3)
O2—H2A···O1^v^	0.82 (2)	2.07 (2)	2.867 (3)	167 (3)
O2—H2B···O1^vi^	0.83 (2)	1.97 (2)	2.806 (3)	178 (3)

Symmetry codes: (ii) *x*, *y*−1, *z*; (iii) −*x*+1, *y*, −*z*+5/2; (i) −*x*+1, *y*, −*z*+3/2; (iv) *x*, −*y*+1, *z*−1/2; (v) −*x*+1/2, *y*+1/2, −*z*+3/2; (vi) −*x*+1/2, −*y*+3/2, −*z*+1.
